# Exploring the relationship between home environmental characteristics and restorative effect through neural activities

**DOI:** 10.3389/fnhum.2023.1201559

**Published:** 2023-09-20

**Authors:** Tao Shen, JunYao Wang, Yingfan Fu

**Affiliations:** ^1^College of Design and Innovation, Tongji University, Shanghai, China; ^2^Academy of Art and Design, GongQing Institute of Science and Technology, Gongqing City, China; ^3^Integrated Design Studies, Universiti Putra Malaysia, Putrajaya, Malaysia; ^4^School of Arts, Universiti Sains Malaysia, Gelugor, Malaysia; ^5^Arts College, Wuyi University, Nanping, China

**Keywords:** interior design, restorative effect, home characteristic, restorative factor, neuroarchitecture

## Abstract

As society and the economy have advanced, the focus of architectural and interior environment design has shifted from practicality to eliciting emotional responses, such as stimulating environments and innovative inclusive designs. Of particular interest is the home environment, as it is best suited for achieving restorative effects, leading to a debate between interior qualities and restorative impact. This study explored the relationships between home characteristics, restorative potential, and neural activities using the Neu-VR. The results of the regression analysis revealed statistically significant relationships between interior properties and restorative potential. We examined each potential characteristic of the home environment that could have a restorative impact and elucidated the environmental characteristics that should be emphasized in residential interior design. These findings contribute evidence-based knowledge for designing therapeutic indoor environments. And combining different restorative potential environments with neural activity, discussed new neuro activities which may predict restorativeness, decoded the new indicators of neuro activity for environmental design.

## 1. Introduction

The home environment, which not only provides a place of residence but is also connected to broader research fields such as restorativeness, kinship, storage, stimulation, intimacy, and productivity (Graham et al., 2015), has been a subject of study for decades. Early research found its connections with physical health (Board, 1949) and mental development (Burks, 1948). To date, the growing number of interdisciplinary studies has formed a burgeoning area because the functions of residence, amenities, and psychological meanings relate to numerous aspects of daily life (Altman and Werner, 2013). Meanwhile, some research highlighted the importance of the home environment and called for concern, such as addressing issues for an aging society (Gitlin, 2003), developing new strategies for treating diseases [e.g., dementia (Gitlin and Corcoran, 1996), chronic disease (Hussain et al., 2015)], and applying smart home technologies for improved life quality (Sovacool and Furszyfer Del Rio, 2020).

Recently, growing scientific concerns have emerged regarding emotional reactions to architectural or interior environments due to the severe neurodivergence and isolation of the global pandemic, such as innovative inclusive designs (Narenthiran et al., 2022) and restorative built settings (Bornioli and Subiza-Pérez, 2022). Regarding restorative environment, it refers to environment can facilitate to restore physical and mental health and has been flourishing (Menardo et al., 2021). With respect to restorative effects in home environments, a study argued that the home may be the most qualified place for restorativeness (Staats, 2012, p. 452). A case of home environment modification supported this viewpoint, finding positive results for health and wellbeing compared to the situation before (Carnemolla and Bridge, 2016). Furthermore, more solid connections between home environment and restorative effects were revealed in the literature. According to investigations into various types of home environmental evaluations, some researchers discovered restorative effects, such as a second home cabin (Bjerke et al., 2006) and a second home in a rural area (Pitkänen et al., 2020). Additionally, the home environment could prevent long-term mental problems and benefit psychological restorativeness for victims of natural disasters (Delgado, 2022), people isolated by the pandemic (Meagher and Cheadle, 2020), and immigrants (Wood and Martin, 2020). From these previous studies, we found connections between home environment and restorativeness, and showed evidence of home restorativeness.

Home restorativeness encompasses not only the holistic atmosphere of the home environment but also specific environmental characteristics. From the history of restorative experiences, the element of nature is essential, as numerous studies have discussed the positive effects of natural environments on aspects such as psychophysiological status (Ulrich, 1981), attention restoration (Hartig et al., 1991), and cognitive benefits (Berman et al., 2008). Consequently, incorporating nature into interior design may facilitate the restoration of adverse symptoms (Craig et al., 2022). For example, natural elements in window views (Kaplan, 2001), window views of the sky (Masoudinejad and Hartig, 2020), natural lighting (Abdelaziz Mahmoud et al., 2023), indoor plants (Shibata and Suzuki, 2001), and private gardens at doorsteps (Stigsdotter and Grahn, 2004) have all been found to have restorative effects on residents. In addition to visual perception, the acoustics of natural sounds can help people connect with nature and foster psychological stress restoration (Alvarsson et al., 2010), as well as improve self-rated health conditions (Dzhambov et al., 2021). Furthermore, the incorporation of natural materials in interiors can benefit individuals. Wood, a commonly used interior material, can evoke bright and pleasant emotions, and convey warm feeling (Watchman et al., 2017) and has demonstrated potential roles in stress restoration (Burnard and Kutnar, 2015).

Interior characteristics and restorativeness extend beyond the mere implementation of natural elements. A study conducted in Scotland discovered that housing fixtures, overcrowding, dwelling type, and access to gardens were linked to residents’ anxiety and self-assessed health issues (Macintyre et al., 2003). For patients’ rooms, warm colors and larger spaces with greater width-length ratios might enhance restorative quality (Gao and Zhang, 2020). For rooms intended for the elderly, accessibility is a crucial factor due to its connection with health-related concepts (Stineman et al., 2011). At a smaller scale, furniture design (Dazkir and Read, 2012), artwork (Nejati et al., 2016), and lighting patterns (Abboushi et al., 2019) have demonstrated links to restorativeness. Additionally, waterscapes, often overlooked in buildings, play an unexpectedly significant role in restorativeness, including both in-building waterscapes (White et al., 2010) and sea views (Nang Li et al., 2012).

It is evident that environmental characteristics are associated with restorative performance based on existing literature. However, many studies rely on subjective data forms (Table 1), which may introduce biases due to subjective decision-making or estimation processes (Tversky and Kahneman, 1974). Rad et al. (2021) suggested improving design by focusing on human preferences and employing neuroscientific insights instead of subjective feedback in architecture to develop more suitable and universal designs for neuro-architecture. Consequently, with the advancement of virtual reality (VR) and neurotechnology, neuroarchitecture, which measures environmental effects by recording neurophysiological data (Edelstein and Macagno, 2012), has emerged as a new discipline with a promising framework in the environmental design field (Bower et al., 2019; Karakas and Yildiz, 2020; Higuera-Trujillo et al., 2021). This approach enables in-depth environmental design studies through the analysis and discussion of neurophysiological data, such as examining design variables for stress reduction in hospital waiting rooms (Higuera-Trujillo et al., 2020). In this study, we aimed to identify relations between interior characteristics and restorative potential, and neural activities, to facilitate an in-depth and comprehensive exploration of home environmental restorativeness by examining the relationship among these three variables. Based on this main research aim, several subsidiary issues were discussed in this study:

**TABLE 1:** Interior characteristic and restorativeness.

User	Interior characteristic	Restorative effect aspect	Data form	References
People who is sheltering-in-place or telework	Implementing nature in the interior	Stress	Attention task, skin conductance, salivary alpha-amylase, subjective stress report, heart rate, blood pressure, subjective mood, self-reported satisfaction, and physiological responses	Craig et al., 2022
Apartment resident	Nature content in window view	Satisfaction and wellbeing	Questionnaire	Kaplan, 2001
Densely populated cities’ resident	Window view to sky	Attention restoration	Questionnaire	Masoudinejad and Hartig, 2020
—	Natural lighting	Psychophysiological well being	N/A	Abdelaziz Mahmoud et al., 2023
—	Plant in room	Mental fatigue	Questionnaire	Shibata and Suzuki, 2001
—	Private garden at doorstep	Stress	Questionnaire	Stigsdotter and Grahn, 2004
—	Natural sounds	Psychological stressor Self-rated health	Skin conductance	Alvarsson et al., 2010; Dzhambov et al., 2021
—	Wood material	Bright, pleasant, warm Stress	Questionnaire, autonomic nervous system, and hypothalamic–pituitary–adrenocortical axis of the endocrine system	Burnard and Kutnar, 2015; Watchman et al., 2017
—	Housing fixtures, overcrowding, dwelling type, access to garden	Anxiety and self-assessed health problem	Questionnaire	Macintyre et al., 2003
Patient	Warm color, bigger rooms with a large width-length ratio	Patient restorative quality	Questionnaire and skin conductance	Gao and Zhang, 2020
Elderly People	Accessibility	Perceived health, dementia	Interview	Stineman et al., 2011
Hospital staff	Artwork Access to outdoor space	Restorative qualities	Questionnaire	Nejati et al., 2016
—	Curvilinear forms of furniture	Pleasant-unarousing emotions	Questionnaire	Dazkir and Read, 2012
—	Light pattern	Relaxation and excitement,	Questionnaire	Abboushi et al., 2019
—	Waterscape Sea view	Mental fatigue Noise annoyance	Questionnaire Questionnaire	White et al., 2010; Nang Li et al., 2012

• To find the home characteristics which may convey the positive impacts on restorative potential.
• To explore the connections between restorative potential and neural activities, discuss the neural indicator for restorativeness.

## 2. Materials and methods

### 2.1. Experimental design

We chose modeling home environments from a model base,^1^ which hosts numerous building environmental 3D models and serves as a primary platform for designers to share and exchange ideas. The model base features 16 different interior design styles (Chinese, European, Simple European, Mediterranean, Modern, Industrial, American, Japanese and Korean, New Chinese, New classical, European classical, Northern Europe, French, Post-modern, Light luxury, and Southeast Asian). We selected the most recommended living room and bedroom models for each style, resulting in a total of 32 models for this investigation. These models represent popular styles and home design images frequently used in China.

The experiment was divided into two parts. First, participants were asked to rate the restorative potential of each selected environment using a questionnaire comprising a set of restorative component rating scales. After measuring the degrees of environmental characteristics, we used regression analysis (Liang and Zeger, 1993) to determine the relationships between home characteristics and restorative potential. In the second part of the experiment, we selected the environments with the highest and lowest scores based on the restorative component rating results and tested neuro reactions to these two environments to show the connections between restorativeness and neuro activities. This two-part approach might afford an in-depth discussion of the relationships among home environmental characteristics, restorativeness, and neuro activities.

### 2.2. Measures and participants

To measure the restorative potential, we used the questionnaire developed by Laumann et al. (2001) to evaluate the restorative components of an environment based on Kaplan and Kaplan’s (1989) restorative theory in this study. The questionnaire included 22 rating scales and was posted on a web platform^2^ along with pictures of all modeling environments. A total of 66 participants (22 males and 44 females, *M* = 23 years old), all from a Chinese college, completed the questionnaire.

Regards to home characteristics, we identified potential elements related to restorativeness from the literature. Celikors and Wells (2022) found that a small portion of restorative qualities was associated with low-level visual features, but the potential and limits of this feature for restorativeness remained unknown. In a residential streetscapes study, Lindal and Hartig (2013) found that the effect of building height on restorativeness was partially mediated. Lee et al. (2012) discovered that some fabrics could extract semantic scales of nature, joyfulness, softness, etc. Combining these three potential characteristics with the features we summarized earlier, we established a set of 15 environmental characteristic scales (Table 2). Then, we invited two associate professors with 15 years of teaching and working experience in the interior design field to discuss and evaluate the degree of these characteristics as shown in each selected home design model picture (the Cohen’s kappa coefficient = 0.957). This expert evaluation helped us better understand the relationships between home characteristics and restorative potential and provided insights for the next phase of the study, which involved analyzing neuro reactions in the best and worst scoring environments.

**TABLE 2:** Potential restorative characteristics for home environment.

	Characteristic	References
1	Overall window view	Kaplan, 2001; Masoudinejad and Hartig, 2020; Abdelaziz Mahmoud et al., 2023
2	Nature view	
3	Sky view	
4	Window size	
5	Greenness	Shibata and Suzuki, 2001
6	Wood material	Burnard and Kutnar, 2015; Watchman et al., 2017
7	Warm colors	Gao and Zhang, 2020
8	Cold colors	
9	Room size	Gao and Zhang, 2020
10	Furniture form	Dazkir and Read, 2012
11	Artwork	Nejati et al., 2016
12	Access to outdoor space	
13	Visual feature	Celikors and Wells, 2022
14	Height	Lindal and Hartig, 2013
15	Textile/fabric	Lee et al., 2012

The application of Neu-VR in this study allowed researchers to collect objective data on the nervous reactions of participants when they experienced various home environments. By monitoring cerebral hemoglobin concentration, pupil radius, attention shift frequency, blink frequency, and eye gaze position, the study was able to gain valuable insights into the participants’ neurophysiological responses to the environments. 15 participants (5 females and 10 males, *M* = 28 years old) participated in this phase of the study. The data collected through Neu-VR complemented the subjective feedback obtained from the questionnaire, providing a more comprehensive understanding of the relationships between home environmental characteristics, restorativeness, and neuro activities.

By combining the results from both the questionnaire and the neurophysiological data obtained through Neu-VR, the study aimed to create a more holistic view of the factors that contribute to restorative home environments. The findings can then be used to inform future interior design and architectural practices, ultimately creating more restorative and beneficial spaces for occupants.

## 3. Result

### 3.1. Home characteristic and restorative potential

Initially, the zero-order correlation analysis method was employed to identify the relationship between home characteristic ratings and restorative potential (Table 3). In this investigation, three types of view characteristics were considered: overall window view, natural view, and sky view. The results of the correlation analysis revealed no significant association between natural window view and restorative potential. However, sky view, overall window view, and window size were found to be related to restorative potential. Regarding the color categories assessed in each home image, both warm and cool colors were evaluated. Factors such as greenness, warm colors, room size, and access to outdoor space demonstrated significant relationships with the restorative potential of homes. In contrast, wood material, cool colors, furniture form, artwork, visual features, room height, and textile materials did not show any correlation with restorative potential.

**TABLE 3:** Correlations of restorative potential with each home characteristic.

	1	2	3	4	5	6	7	8	9	10	11	12	13	14	15
1. Overall window view	–														
2. Natural view	0.56*	–													
3. Sky view	0.79*	0.54*	–												
4. Window size	0.71*	0.38*	0.47*	–											
5. Greenness	0.51*	0.73*	0.36*	0.20	–										
6. Wood material	0.05	−0.03	0.15	−0.13	0.17	–									
7. Warm colors	0.17	0.30	0.35	−0.07	0.27	0.49*	–								
8. Cold colors	−0.16	−0.08	−0.21	0.06	−0.08	−0.38*	−0.26	–							
9. Room size	0.40*	0.14	0.25	0.42*	0.23	0.14	0.19	−0.24	–						
10. Furniture form	−0.17	−0.36*	−0.04	−0.25	−0.23	0.09	−0.15	−0.09	−0.12	–					
11. Artwork	0.06	−0.24	0.08	−0.05	−0.16	−0.10	0.17	0.10	−0.18	−0.06	–				
12. Access to outdoor space	0.71*	0.54*	0.55*	0.44*	0.56*	0.23	0.31	−0.31	0.39*	−0.27	−0.16	–			
13. Visual feature	0.00	0.10	−0.10	0.01	0.33	0.08	−0.09	−0.02	0.22	−0.18	−0.07	0.16	–		
14. Height	0.00	0.25	0.03	−0.05	0.24	0.38*	0.31	−0.17	0.49*	−0.18	−0.23	0.23	0.51*	–	
15. Textile/fabric	−0.05	−0.07	−0.07	−0.03	−0.15	−0.26	−0.39*	0.20	−0.46*	−0.13	0.08	−0.09	−0.12	−0.51*	–
Restorative potential	0.72*	0.30	0.58*	0.41*	0.37*	0.21	0.35*	−0.25	0.56*	−0.04	0.02	0.81*	0.10	0.25	−0.10

*p < 0.05.

To identify home characteristics that can predict restorative potential, we employed backward regression to eliminate characteristics that did not have a significant impact. The analysis yielded a model with a high confidence interval of *p* < 0.001 (*R*^2^ = 0.843, *F* = 18.397) (Table 4). Considering the regression coefficients and significance levels, four predictors of home environment were identified as significant characteristics (*p* < 0.05) that can predict restorative potential: overall window view, warm colors, room size, and access to outdoor space. These characteristics were all positively associated with restorative potential, indicating that enhancements in these home characteristics are perceived to increase restorative potential.

**TABLE 4:** Regression of home characteristics with restorative potential.

Predictor	*B*	*Beta*	*t*	*Sig.*
Overall window view	0.041	0.311	2.437	0.023
Warm colors	0.041	0.221	2.326	0.029
Room size	0.070	0.297	2.796	0.010
Access to outdoor space	0.070	0.579	4.650	0.000

### 3.2. Neuro data

**FIGURE 1:**
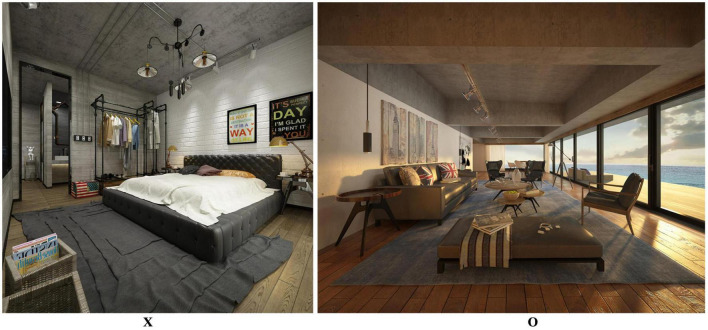
Pictures of home environments with the worst (X) and best (O) restorative potential scores. Reproduced with permission from (MoxingYun), available at http://www.moxingyun.com/shinei/3d-sn18816760.html and http://www.moxingyun.com/shinei/3d-sn18816442.html.

We asked the 15 participants to experience two home environments with the worst (X) and best (O) restorative potential scores in Neu-VR (Figure 1). They experienced both environments in a random order, with each exposure lasting 3 min. The data for cerebral hemoglobin density, pupil radius, attention shift frequency, blink frequency, and eye gaze position were recorded by the Neu-VR sensors. After conducting a paired sample *t*-test analysis, we obtained the results for differences in pupil radius, attention shift frequency, blink frequency, and cerebral hemoglobin density at four positions (Table 5).

**TABLE 5:** Paired sample *T* test result of Neuro data in two home environments of the worst and best restorative potential scores.

Paired samples statistics						Paired samples correlations		Paired samples test			
	Neuro item	Mean	N	Std. deviation	Std. error mean	Correlation	Significance (Two-sided p)	Mean	95% Confidence interval of the difference		Significance (Two-sided p)
									Lower	Upper	
Pair 1	Pupil Radius (millimeters)_O	1.82	15	0.17	0.05	0.76	**0.00**	−0.31	−0.38	−0.23	**0.00**
	Pupil Radius (millimeters)_X	2.13	15	0.21	0.05						
Pair 2	User Attention Shift (every second)_O	9.26	15	2.25	0.58	0.68	**0.01**	−1.60	−2.71	−0.49	**0.01**
	User Attention Shift (every second)_X	10.86	15	2.65	0.68						
Pair 3	Blink (every minute)_O	26.76	15	12.04	3.11	0.39	0.16	−13.40	−22.15	−4.65	**0.01**
	Blink (every minute)_X	40.16	15	15.88	4.10						
Pair 4	Hb Density (L1 cm)_O	0.15	15	0.17	0.04	−0.48	0.07	0.02	−0.14	0.18	0.79
	Hb Density (L1 cm)_X	0.13	15	0.18	0.05						
Pair 5	Hb Density (L3 cm)_O	0.28	15	0.24	0.06	−0.25	0.37	0.20	−0.01	0.41	0.06
	Hb Density (L3 cm)_X	0.08	15	0.24	0.06						
Pair 6	Hb Density (R1 cm)_O	0.07	15	0.17	0.04	0.12	0.66	−0.03	−0.20	0.13	0.68
	Hb Density (R1 cm)_X	0.10	15	0.26	0.07						
Pair 7	Hb Density (R3 cm)_O	0.25	15	0.22	0.06	0.15	0.59	0.18	0.01	0.34	**0.04**
	Hb Density (R3 cm)_X	0.08	15	0.23	0.06						

The mean value of pupil radius for environments O and X were 1.82 ± 0.17 and 2.13 ± 0.21 mm, respectively. Environment O exhibited a pupil radius 0.31 less than environment X (95% Confidence Interval: −0.38 to −0.23), with the difference being statistically significant (*p* < 0.001). The mean value of attention shift frequency for environments O and X were 9.26 ± 2.25 and 10.86 ± 2.65 times, respectively. Environment O exhibited an attention shift frequency 1.60 less than environment X (95% Confidence Interval: −2.71 to −0.49), with the difference being statistically significant (*p* = 0.01). Although there were statistical differences between blink pairs (*p* = 0.01) and hemoglobin density at position R3 (*p* = 0.04), their *p*-values of correlation were greater than 0.05. No statistical differences were observed between pairs of hemoglobin density at positions L1, L3, and R1.

## 4. Discussion

### 4.1. Restorative factors

This study is among the few that explore home characteristics and restorativeness using both subjective and objective data. The results identified several home characteristics as strong predictors of restorativeness. Firstly, our findings from the regression analysis indicated that window view is statistically associated with home restorativeness, which is consistent with previous research on window views. Numerous benefits of window views have been documented, such as opportunities for restorativeness, residential satisfaction, and wellbeing (Kaplan, 2001). Additionally, window views of green spaces have been shown to have relaxing effects and reduce stress levels (Elsadek et al., 2020). Sky views have been found to have the highest restorative potential in urban contexts compared to views of people and street trees (Masoudinejad and Hartig, 2020). Other positive effects of window views include work ability and satisfaction (Lottrup et al., 2015), thermal comfort and cognitive performance (Ko et al., 2020). In contrast, office environments without window views can induce tension and anxiety, as evidenced by electromyography (EMG), electroencephalography (EEG), and blood volume pulse (BVP) data (Chang and Chen, 2005).

In addition to mental benefits, window views have been linked to potential physical recovery, such as decreased negative evaluative comments from nurses for surgery patients (Ulrich, 1984), improvement of self-reported physical health for coronary and pulmonary patients (Raanaas et al., 2012), and reduced usage of analgesics and pain relief for cesarean women (Wang et al., 2019). Moreover, windows are associated with another restorative factor, natural lighting, which is considered the best light source (Abdelaziz Mahmoud et al., 2023). As different angles and ratios can lead to various views, window design for restorativeness may involve multiple dimensions. Our correlation results showed a significant relationship between window size and restorative potential, although no significant effect was found in the regression analysis. Some researchers have discussed the effects of window size in terms of minimum window size design (Ne’eman and Hopkinson, 1970), energy balance (Persson et al., 2006), and glare of sunlight presence (Boubekri and Boyer, 1992). To determine whether larger windows are more restorative, further research is needed to investigate the correlations between specific designs. Consequently, several studies have concluded that window views are associated with psychophysiological wellbeing for residents and have proposed multi-criteria evaluation methods for window design (Ko et al., 2022; Lin et al., 2022). And the virtual window view was also discussed (Radikovic et al., 2005).

The second element was warm color, which is consistent with the design of patient ward where inpatients preferred warm colors over cold or white (Gao and Zhang, 2020). According to the extent element of restoration theory, environmental components can easily occupy an occupant’s mind (Herzog et al., 2003). Warm colors exhibited excellent attention-guiding qualities (Pal et al., 2012), which may explain their use in learning materials designed to evoke positive emotions and enhance understanding during media learning (Plass et al., 2014; Münchow et al., 2017). Color and mood are interconnected concepts (Valdez and Mehrabian, 1994), and even colors within the same scheme can elicit different emotional responses (Naz and Epps, 2004). For restorativeness, light warm colors are recommended based on findings that pale colors evoked feelings of relaxation, calmness, and pleasantness (Al-Ayash et al., 2016) and that light colors were more likely to elicit positive moods such as joy and relaxation (Jonauskaite et al., 2019).

The third characteristic was room size, which is associated with various aspects of daily life, including emotional response to sound (Tajadura-Jiménez et al., 2010), air quality (Cherrie et al., 2011), and self-reported stress (Sundstrom, 1975). And room size directly impacts physical openness and spatial quality (Franz et al., 2005). Regarding room size and restorativeness, Gao and Zhang (2020) found that larger rooms may facilitate patient recovery. Nehrke et al. (1979) suggested that room size may influence health status, as small rooms can convey a sense of limitation (Meyers-Levy and Rui, 2007) and exacerbate small-space stressors such as lighting, noise, vibration, radiation, and low air quality (Li et al., 2022). These stressors can be magnified in long-term isolation, leading to sleep disorders and other adverse effects (Meng et al., 2020; Palinkas and Suedfeld, 2021). These findings emphasize the importance of room size in designing a restorative home environment.

The final characteristic was access to outdoor space. Nejati et al. (2016) found that rooms with access to outdoor space had significantly greater restorative potential than those without. This element is linked to other restorative cases, such as cabins (Bjerke et al., 2006) and rural second homes (Pitkänen et al., 2020), where offer easy access to outdoor spaces. Additionally, private gardens at the doorstep have been shown to reduce stress (Stigsdotter and Grahn, 2004). Numerous studies highlight the importance of access to outdoor spaces for all age groups (Mapes, 2010; DEFRA, 2011). Tremblay et al. (2015) stated that outdoor spaces are crucial for children’s health, as they encourage more movement, less sitting, and longer playtimes, lowering obesity risk (Porter et al., 2018) and promoting positive mental health (Hinkley et al., 2018). Compared to indoor physical activity, exercising outdoors has been shown to alleviate tension, anger, and depression better (Thompson Coon et al., 2011). Long-term outdoor activity benefits have also been observed in Positive Youth Development programs (Armour and Sandford, 2013). Consequently, access to green outdoor spaces in the workplace has been shown to improve worker wellbeing and reduce stress (Lottrup et al., 2013).

### 4.2. Neuro data

Upon obtaining the results for each environment’s restorative potential score, both the environments with the highest and lowest restorative potential scores were input into the Neu-VR system to evaluate participants’ reactions. The difference in pupil radius between environments O and X was 0.31 mm, a statistically significant value. Pupillometry has shown its history and validity of measuring active state of neocortex (Larsen and Waters, 2018) and neurobiologists found the associations between restorative environment and brain activity (Martínez-Soto et al., 2013). Beatty (1982) observed a pupil dilation of approximately 0.3 mm when comparing relaxed and cognitively-loaded conditions. The 0.31 mm difference between the two environments surpasses the 0.3 mm threshold. Although pupil size is predominantly influenced by light luminance (De Groot and Gebhard, 1952) and visual environment (Larsen and Waters, 2018), the possibility of using pupil size to measure restorativeness should be considered, given that light is a factor in restorativeness. Further examination of this potential indicator is warranted.

A statistically significant difference was observed in recorded attention shift frequency between environments O and X, with the former exhibiting a lower frequency of 1.60 shifts per second. According to Neu-VR equipment specifications, attention shift frequency corresponds to the frequency of visual focus changes. This concept is analogous to the fixation-click count employed in visual monitoring research to evaluate visual attention in air traffic controller training (Wee et al., 2020). Fascination, a component of Attention Restoration Theory, posits that a captivating environment effortlessly holds an individual’s attention (Kaplan and Kaplan, 1989). And eye movement as effective an interference (Lawrence et al., 2004), consequently, lower attention shift frequencies may indicate a more restorative environment. Further testing with robust restorative environment designs is required to validate this predictor. Despite the statistical differences in blink and hemoglobin density measures, correlations between these metrics suggest the presence of other influencing factors. As such, they cannot be utilized as reliable indicators of environmental restorativeness.

**FIGURE 2:**
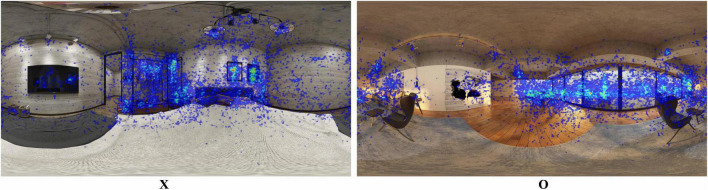
Gaze position heatmap of the home environment with worst restorative potential score (X) and home environment with best restorative potential score (O). Reproduced with permission from MoxingYun, available at http://www.moxingyun.com/shinei/3d-sn18816760.html and http://www.moxingyun.com/shinei/3d-sn18816442.html.

Gaze position data were collected, and heatmaps for environments X and O were generated. Figure 2 (X) displayed dispersed gaze positions, with a significant proportion focused on the metal frame and textual artwork. One hypothesis posited that metal may convey negative effects on restorativeness due to its association with adverse outcomes [e.g., unhealthy, enclosed, and depressive imagery of white steel (Sakuragawa et al., 2005); cold, industrial, and unpleasant connotations of steel (Wastiels et al., 2013)]. Further research with objective data is needed to investigate this hypothesis. In contrast, Figure 2 (O) revealed centralized gaze positions on window views and artworks, consistent with the high perceived restorativeness of seascapes (Nang Li et al., 2012) and artwork (Nejati et al., 2016).

## 5. Conclusion and limitation

This study identified four key characteristics essential for designing a restorative home environment, including favorable window views, light warm colors, spacious room sizes, and outdoor access. Although the window design is driven by multicriteria, interior designers and architects could consider the rule that something is better than nothing for designing a restorative home. While both pupil radius and attention shift displayed statistically significant differences between the best and worst restorative environments, further research is necessary to validate the reliability and applicability of these two potential measures.

This study explored the relationships between home characteristics restorativeness, and Neu-VR data for measuring restorativeness. Several limitations were identified concerning the methodology and results. Firstly, increasing the sample size for the two experiments would enhance the validity of the findings in demonstrating the relationships between restorative potential and environmental characteristics, restorativeness and neuro-data. Secondly, different regions’ participants may have different attitudes to the same design style. region variation was not considered in this investigation because of all Chinese participants. Thirdly, no prior literature provided valid examples of pupil radius, attention shift frequency, and restorativeness in relation to neuro-data. Although the results showed potential, further research is required to investigate these predictors. Lastly, regarding negative restorativeness characteristics, although the heatmap of gaze positions offered a hypothesis, low restorative potential designs were not examined. The study’s novelty lies in its attempt to investigate the relationships between home characteristics, restorative potential, and neural activities using Neu-VR. Four environmental characteristics, that should be emphasized in residential interior design, were found to predict home restorativeness, and two neurodata types showed potential for expressing restorativeness. These findings cement evidence-based knowledge for designing restorative home environments and contribute to new measures of neuro activity for environmental design.

## Data availability statement

The raw data supporting the conclusions of this article will be made available by the authors, without undue reservation.

## Ethics statement

The studies involving humans were approved by the Japan Advanced Institute of Science and Technology. The studies were conducted in accordance with the local legislation and institutional requirements. The participants provided their written informed consent to participate in this study.

## Author contributions

TS performed the literature research and draft the manuscript, reviewed and selected the articles to include in the systematic review, and performed the manuscript supervision and project administration. YF and JW revised the drafts. All authors contributed to the conception and design of the work and have read and agreed to the published version of the manuscript.

## Footnotes

1
http://www.moxingyun.com/

2
https://www.wjx.cn/
